# Two-Dimensional Phononic Crystal Based Sensor for Characterization of Mixtures and Heterogeneous Liquids

**DOI:** 10.3390/s22072816

**Published:** 2022-04-06

**Authors:** Nikolay Mukhin, Mykhailo Kutia, Alexander Aman, Ulrike Steinmann, Ralf Lucklum

**Affiliations:** 1 Institute for Automation Technology, Otto von Guericke University of Magdeburg, 39106 Magdeburg, Germany; m.kutia@nanofract.com (M.K.); ulrike.steinmann@ovgu.de (U.S.); ralf.lucklum@ovgu.de (R.L.); 2 NanoFract UG, 39106 Magdeburg, Germany; 3 Otto Vollmann GmbH & Co. KG, 58285 Gevelsberg, Germany; a.aman@vollmann-group.com

**Keywords:** liquid sensor, phononic crystal sensor, metamaterials, transmission spectra, solid–liquid interaction, emulsions

## Abstract

We show new approaches to developing acoustic liquid sensors based on phononic crystals. The proposed phononic crystal integrates fluidic elements. A solid block with periodic cylindrical holes contains a defect—a liquid-filled cylindrical cavity. We pay attention to acoustic excitation and the readout of the axisymmetric cylindrical resonator eigenmode of the liquid-filled defect in the middle of the phononic crystal structure. This mode solves the challenge of mechanical energy losses due to liquid viscosity. We also analyze the coupling effects between oscillations of liquid and solid systems and consider coupling issues between piezoelectric transducers and the liquid-filled cavity resonator. The numerical simulation of the propagation of acoustic waves through the phononic crystal sensor was carried out in COMSOL Multiphysics Software. The phononic crystal was made of stainless steel with mechanically drilled holes and was fabricated for experimental verification. We show that a tuning of the solid–liquid vibrational modes coupling is the key to an enhanced level of sensitivity to liquid properties. Besides (homogeneous) water–propanol mixtures, experimental studies were carried out on (disperse) water–fuel emulsions.

## 1. Introduction

Phononic crystals (PnCs) are currently broadly used to control, direct, and manipulate sound waves. Phononic crystals were introduced approximately thirty years ago [1,2]. There are a great deal of different phononic crystal realizations that accomplish translation symmetry in 2D and 3D designs. Bulk phononic crystals have a periodic modulation in their density and sound velocity for longitudinal and transverse polarization. They create band gaps at so-called Bragg frequencies or wavelengths commensurate to their lattice constant. It is, therefore, an immanent property of a phononic crystal that its geometry can be scaled. The existence of so-called band gaps prevents elastic mechanical wave propagation through solids or acoustic wave propagation through fluids in the corresponding frequency range, at least in certain crystallographic directions. The major efforts in PnC studies have been devoted to the search for absolute band gaps in perforated solids or solid–solid structures. For comparison, the case of solid–liquid structures has been much less studied in practice.

Unlike the bulk wave in the infinite PnC, in finite structures, elastic waves are scattered by large-scale discontinuities such as free surfaces, in addition to the periodically arranged scatters [3]. It should be taken into account when analyzing band gaps of finite PnC structures that the transmission and reflection spectra become dependent on the number of lattice periods and boundaries in the finite structures. Refs. [4,5,6] contain examples of experimental studies of phononic crystals of various designs. For more details on PnCs’ modern designs and concepts, see the latest reviews [7,8].

Understanding and controlling the phononic properties of materials provides opportunities to a large variety of technical applications [9], from earthquake protection, reduction in environmental noise [10], waveguides and filters [11,12], and acoustic diodes [13], to gaining electricity from waste heat [14,15]. We are specifically interested in the exploitation of phononic crystals as sensors, as introduced in [16]. The PnC sensor combines the acoustic resonator concept with the ultrasonic wave propagation concept by introducing a liquid cavity into the propagation path of the ultrasonic wave through a solid phononic crystal [5,17]. In this way, the transmission of ultrasonic waves through the device becomes frequency-dependent when we pour the analyte into the phononic crystal. To be more precise, the sensor is based on the change of a specific parameter—here, the frequency of a single resonance peak within the known transmission spectra of the PnC. The sensor requires that this feature, which is easy to detect, is dependent on the value the applicant is interested in. In many cases, it is composition of a medium (liquid) and the feature employed is a resonance frequency shift together with peak amplitude (or any valued depending on acoustic energy loss in the medium of interest). Liquid cavity resonance especially reflects speed of sound and sound attenuation of the liquid. We further combine both values in a complex number. In the basic sensor concept [5], the phononic crystal sensor platform tailors propagation of ultrasonic waves in a phononic crystal in a manner to excite local resonances inside a liquid-filled cavity. The cavity acts as defect in an otherwise regular structure at frequencies within the band gap of the 2D phononic crystal [5,17,18]. The simplest concept is an ideal 1D cavity, where the fundamental resonance frequency is a measure of the speed of sound of the liquid inside the cavity. Since the speed of sound is the value averaged along the whole path of the acoustic wave inside the cavity, the fundamental resonance frequency consequently represents a volumetric property of the cavity resonator. Behind the primary value speed of sound, many other technologically relevant parameters can be determined, e.g., the concentration of one component of a mixture.

In contrast, the well-known resonant chemical sensors, e.g., the quartz crystal microbalance (QCM), respond to properties of a thin interfacial layer between analyte and sensor, e.g., an adsorbed mass. The resonator device itself (the quartz crystal) is not influenced by the analyte.

The Q-factor of resonance is the key factor defining the resolution of both the frequency and the attenuation measurement. However, neglecting acoustic energy dissipation in materials for a moment, the Q-factor is also determined by the boundary conditions of the resonating element. In the often-assumed ideal case, the surface of the resonator completely reflects the incoming wave, hence at resonance of a 1D cavity of thickness *h*, the wavelength λ = *h*/2 holds. In reality, part of the acoustic wave is transmitted through the cavity surface, specifically at the solid–liquid interface. The realization of optimal boundary conditions is, therefore, one major task of the phononic crystal surrounding the liquid cavity. More generally, the PnC must reduce errors from nonideal reflection and diffraction losses as analyzed, e.g., in [19,20]. A high-Q liquid cavity resonator requires a large acoustic mismatch between liquid and container material. From this viewpoint, the phononic crystal surrounding the liquid-filled cavity increases the impedance mismatch beyond the limits of natural materials within the band gap region [20].

The theoretical predictions and experimental verifications demonstrated on real binary mixtures are in very good agreement. A first practical application on a technical product proved the robustness of the sensor [21]. A conservative estimation of the speed-of-sound measurement accuracy is 1 ms^−1^ using standard electronic equipment and without further data processing.

In our previous work, we used defect modes to realize phononic crystal liquid sensors [22,23,24]. We demonstrated that the peak frequency and amplitude related to the defect mode depends on the (complex) speed of sound of the liquid confined in the defect. The sensitivity of sound velocity measurements based on phononic crystals can compete with well-established ultrasonic principles. However, the PnC sensor can outperform these devices when solving three challenges: a very high fabrication accuracy as well as a reduction in shear viscosity losses and acoustic coupling to the surrounding of the cavity. Whereas we do not deepen the technological aspect, this contribution pays attention to the axisymmetric cylindrical resonator eigenmode of the liquid-filed defect in the center of the phononic crystal structure and its excitation and readout. Regarding liquid properties, this study considers not only homogeneous mixtures, but also inhomogeneous liquids, i.e., dispersions.

## 2. Materials and Methods

**Figure 1:**
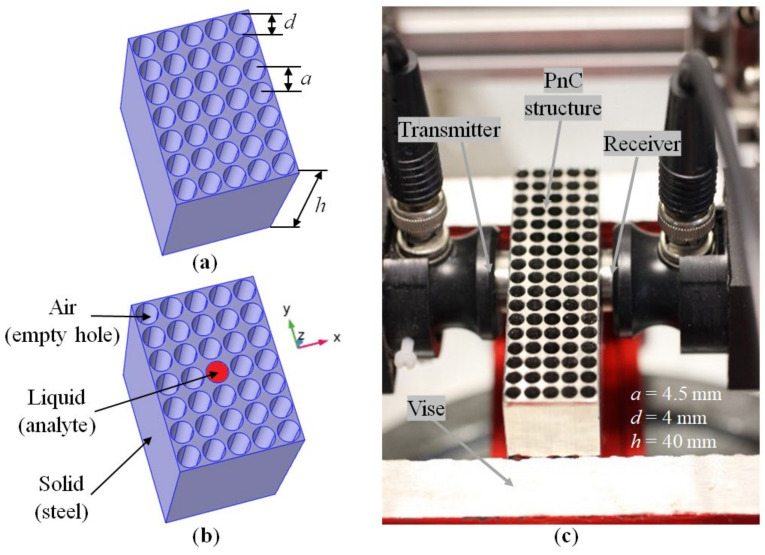
Schematic illustration of a regular phononic crystal (**a**) and a crystal with a point defect (**b**); an experimental sample of a phononic crystal made of stainless steel with the measurement setup for studying its transmission spectra using piezoceramic transducers (**c**).

In this work, the object of study was a two-dimensional phononic crystal, which is a solid-state matrix with a periodic structure of identical cylindrical holes of diameter *d* and spaced apart at a distance *a*, forming a square lattice (Figure 1a). The block, which the phononic crystal was made of, has a considerable height (*h* >> *d*) in order to reduce the influence of boundaries and to bring the properties of the three-dimensional structure closer to a two-dimensional phononic crystal case. Filling the central hole of the PnC with a liquid will lead to a violation of the periodicity of the structure of the crystal lattice. It creates a so-called point defect (Figure 1b). Stainless steel was chosen as the phononic crystal material, since it meets the following requirements: first, the technology is advanced for mechanical processing, specifically for the realization of a periodic system of cylindrical holes; second, high values of the speed of sound and density, which guarantee a high acoustic contrast to the liquid; and third, the material has low mechanical losses.

The theoretical part of the work is related to the study of the spectral characteristics of the regular periodic structure and defect modes. The analysis of band diagrams and transmission spectra was carried out on the basis of numerical simulations using the COMSOL Multiphysics software. The basic equations for the theoretical modeling of PnCs and the boundary conditions used in the numerical calculations are given in the Appendix. To calculate the structure of the vibrational modes of the PnC, the eigenfrequencies of the unit cells with periodic boundary conditions were analyzed. To determine the frequency dependence of the transmission behaviour, a source of longitudinal harmonic acoustic waves was placed on one side of the PnC block, and the response was calculated on the opposite side.

The PnC is a two-dimensional square lattice. The diameter of the holes is 4 mm and the distance between the holes (PnC lattice period) is 4.5 mm. The height of the phononic crystal sample is 40 mm (Figure 1c).

Contact piezoelectric transducers, Panametrics V103-RB (Olympus Corporation, Tokyo, Japan) with a central frequency of 1.0 MHz, were acoustically coupled to the structure (Figure 1c). The Agilent4395A (Agilent Technologies, Hyogo, Japan) network analyzer together with the Agilent 87511A S-parameter extension set (Agilent Technologies, Hyogo, Japan) was used to measure the S21-parameter (transmission) of the structure. S21 is the element of a scattering matrix (or S-matrix used to describe how energy can propagate through a 4-port electric network), here in terms of the forward voltage gain.

Mixtures of water and propanol with well-known properties [25] were used for the experimental study of the defect modes of the phononic crystal structure in order to compare the results with theoretical calculations. By mixing water and propanol, a homogeneous liquid can be obtained with the speed of sound, which varies over a wide range depending on the composition of the mixture. The dependence of the sound speed on the composition is well studied, e.g., [25] and makes the water–propanol mixture a good candidate for testing acoustic sound speed sensors.

A water–fuel emulsion was used as a heterogeneous liquid. We have studied (for the first time in PnCs) water–fuel emulsions since they can improve the performance of internal combustion engines [26,27,28,29,30]. They provide enhanced efficiency and significantly reduce harmful emissions. The general effects behind the use of water–fuel emulsions are as follows: increases the average pressure in the cylinders; accelerates the conversion of CO to CO_2_; increases the completeness of fuel combustion. However, to ensure optimal engine performance, it is necessary to be able to control the state of the water–fuel mixture, the mass fraction of water, and the degree of emulsion degradation.

## 3. Results

### 3.1. Theoretical Calculations of Band Diagrams of Transmission Spectra of a 2D Phononic Crystal with a Liquid-Filled Cavity Defect

The results of modeling the band diagram of a regular periodic structure of a phononic crystal (PnC) as well as the propagation of acoustic waves through a finite structure of the PnC with a point defect are shown below. The simulations were performed by numerical solving the equations for the propagation of acoustic waves through solid and liquid media (see Appendix A).

**Figure 2:**
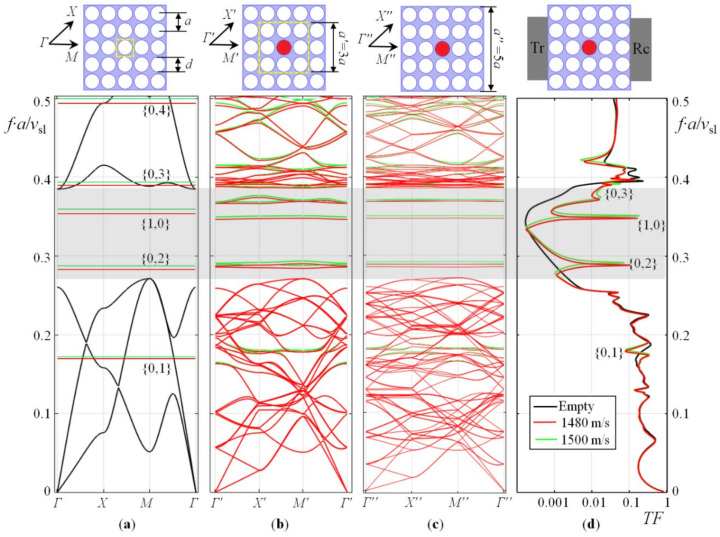
Band diagram for 2D infinite PnC (black curves) and isolated liquid-filled hole resonator eigenmodes for two different speed of sound values (green and red lines) (**a**); Band diagrams for 2D supercells PnC with a liquid-filled central hole for two different speed of sound values (green and red curves) with the lattice constants of *a*′ = 3*a* (**b**) and *a*″ = 5*a* (**c**); Transmission spectrum of the regular finite PnC and the PnC with a liquid-filled hole defect for the two different speeds of sound (**d**). *TF* is the overall acoustic wave transmission factor. The PnC filling factor, defined as π*d*^2^/(4*a*^2^), is 0.62.

Figure 2a shows the band diagram of a two-dimensional infinite PnC made of steel with a periodic arrangement of cylindrical empty holes (black curves). In the same diagram, red and green lines show eigenfrequencies of an ideal cylindrical resonator (IR) filled with a liquid and having completely reflecting walls. For the two-dimensional case, the resonance modes of the cylindrical resonator are numbered by two indices, according to the equation [31]:

(1) $$p_{l,m}\left(r,\theta \right)=p_{l,m}J_{m}\left(\xi_{l,m}^{}r\right)cos\left(m\theta -\pi /2\right);f_{l,m}=\frac{c}{2\pi }\xi_{l,m}^{},$$

where *l* and *m* are integers ≥ 0; *P* is the pressure magnitude, *J* is the Bessel function; the parameter values ξ_*l*,*m*_ can be found from the numerical solution of Equation (2):

(2) $$J_{m}\left(\xi_{l,m}d/2\right)/J_{m+1}\left(\xi_{l,m}d/2\right)=\left(\xi_{l,m}d/2\right)/\left(m+1\right).$$

Parameters *l* and *m* in Equations (1) and (2) number radial (axisymmetric) and azimuthal eigenmodes.

It is important to note that eigenfrequencies of an ideal two-dimensional steel/vacuum phononic crystal and an ideal two-dimensional cylindrical resonator with a liquid have the same hole diameters and are calculated independently; that is, the liquid and solid systems do not interact with each other.

In Figure 2b–d, this interaction was turned on. The central hole of the PnC is filled with a liquid. The band diagram in Figure 2b was calculated for a translational supercell with 3*a* side. Since the size of the unit cell of the PnC has changed, the characteristic points of the first Brillouin zone have changed their coordinates, so they are marked with letters with a prime. The band diagram in Figure 2c is calculated for an enlarged supercell with a size of 5a. The coordinates of points of special symmetry in the first Brillouin zone are determined as follows: *Γ*(0,0), *X*(π/*a*,0), *M*(π/*a*,π/*a*). For the two supercells: *Γ*′(0,0), *X*′(π/*a*′,0), *M*′(π/*a*′,π/*a*′) and *Γ*″(0,0), *X*″(π/*a*″,0), *M*″(π/*a*″,π/*a*″), respectively, where *a*′ = 3*a* and *a″* = 5*a*. In Figure 2, the frequency axis is normalized by multiplying by the lattice constant and dividing by the longitudinal speed of sound in solid (*v*_sl_). This normalized frequency axis reflects the scaling principle of PnCs.

A comparison of Figure 2a–c provides an idea of the formation of defect modes inside the band gap of the PnC (shown by the gray canvas). Moreover, the inclusion of the interaction between the solid and liquid subsystems led to a shift in the eigenfrequencies of the liquid-filled hole resonator, and now these frequencies do not exactly correspond to Equation (1). Calculations show that an increase in the number of periods of a phononic crystal surrounding a defect straightens the dispersion of defect modes (compare Figure 2b,c) and makes them more isolated from the vibrations of the solid matrix. They bring the properties of the defect closer to those of an ideal liquid resonator; however, this is true for modes located in the band gap away from its edges.

Figure 2d shows the result of calculating the transmission spectrum of the finite PnC structure placed between the transmitter (Tr) and the receiver (Rc) of the acoustic waves. Figure 2d shows that the finite structure has a frequency band, within which the transmission is suppressed corresponding to the position of the band gap. The acoustic wave can pass the PnC in the band gap region only at the eigenfrequencies of the defect modes. The design of the PnC was realized in a way that the transmission peak associated with the axisymmetric {1,0} mode of liquid pressure in a cylindrical hole lies in the region of reduced transmission. For phononic crystal sensor applications, the eigenfrequency of this vibrational mode must fall into the band gap for a wide range of liquid compositions, in our case, water, alcohol and their mixtures as well as mixtures of water and liquid hydrocarbons. In addition to this mode, which is the most interesting for the sensor, the second azimuthal {0,2} mode closest to it in frequency can fall into the band gap region.

**Figure 3:**
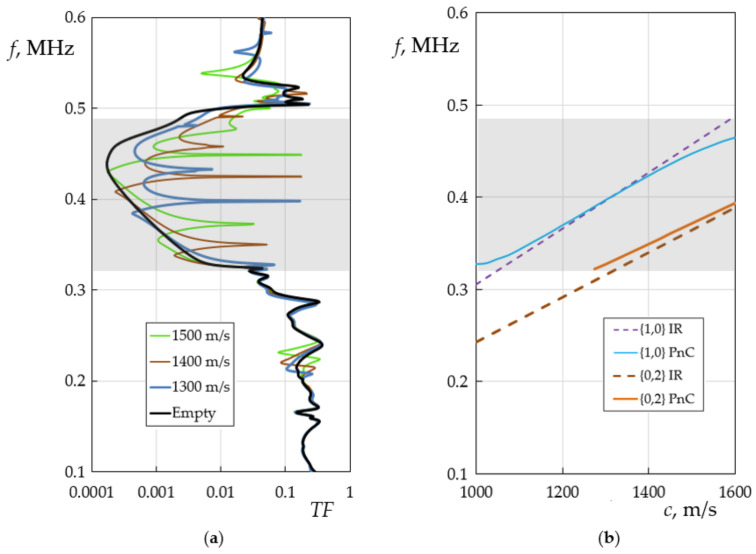
Theoretical transmission spectra of 2D PnC with a defect for different liquid sound velocities (**a**) and the dependence of the transmission peaks on the liquid speed of sound (*c*, m/s) (**b**), corresponding to {1,0} and {0,2} modes for the ideal resonator (IR) and the PnC.

The transmission spectra of the PnC with a defect and the dependence of the resonant frequencies for the {1,0} and {0,2} modes were calculated in a wide range of changes in the sound velocities of liquid (*c*) and shown in Figure 3 on the usual frequency axis in Hertz. The simulation results prove that different localized defect eigenmodes can be exited inside the acoustic bandgap of PnC, which are sensitive to changes in the speed of sound of the liquid. Far from the edges of the bandgaps, the dependence of the resonance frequencies of these modes on the speed of sound is linear, as in an ideal resonator. Closer to the edges of the bandgap, nonlinearities arise (Figure 3b). The nonlinear effect reduces the sensitivity to the speed of sound; therefore, the resonances of the defect modes should be tuned by placing them deeper in the bandgap center of the phononic crystal. Taking into account thermo-viscous losses at the liquid–solid interface using the Navier–Stokes equations [32,33] gives different heights and quality (Q)-factors of peaks for different defect modes. Figure 2d and Figure 3a show that the peak amplitude of the {1,0} mode is much higher and has a higher frequency. This theoretical result has been experimentally verified below.

**Figure 4:**
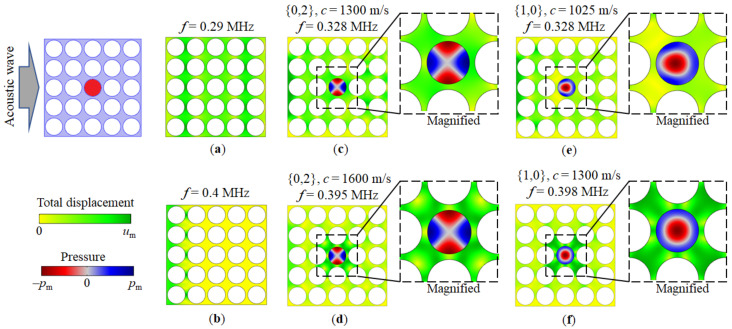
Distribution of mechanical displacement for the PnC with empty holes within the pass band (**a**) and the band gap (**b**), as well as displacement and pressure field distributions for the PnC with a liquid-filled hole defect {0,2} mode (**c**,**d**) and {1,0} (**e**,**f**) mode excitation. The sound velocities of the liquid analyte were chosen in such a way that the resonant frequencies of the vibrational defect modes fall close to the edge (**c**,**e**) or in the middle (**d**,**f**) of the band gap. For clarity, inserts are added with magnified areas around the liquid-filled hole PnC defect.

Figure 4 shows, for comparison, the two cases of the distribution of mechanical displacement fields for the PnC with a regular structure of empty holes (Figure 4a,b) as well as the one with the central hole filled with a liquid (Figure 4c–f). In the first case (Figure 4a), it can be seen that the acoustic wave passes through the PnC at the frequency of the transmission zone. Figure 4b proves that the structure reflects an acoustic wave incident from the left side onto the PnC at a frequency inside the band gap. In the second case, the two resonances are excited in the defect hole (Figure 4c–f) in the middle of the PnC sample. The distribution of liquid pressure in the defect hole corresponds to the second azimuthal {0,2} (Figure 4c,d) and the axisymmetric {1,0} (Figure 4e,f) modes. In one case, the sound velocities of the liquid are chosen so that the resonant frequencies of the vibrational modes of the defect are near the band gap edge (Figure 4c,e). In the second case, they fall into the middle of the band gap (Figure 4e,f).

Comparing the distributions of the pressure fields in Figure 4c,d as well as in Figure 4e,f, it can be noted that for both {0.2} and {1.0} modes excited at frequencies corresponding to the middle of the bandgap, pressure distributions look symmetrical and close to the mode structure in an ideal cylindrical resonator. By contrast, the mode structure exhibits distortions at resonant frequencies close to the band gap edge and the pressure field pattern looks “deformed”. This feature correlates with the linear and nonlinear behaviour of the dependences of the resonant frequencies of the {0.2} and {1.0} modes on the speed of sound in Figure 3b in the middle and close to the edge of the band gap, respectively. Additional considerations for the PnC sensor structure design and sensitivity are given in Appendix B.

### 3.2. Experiments with a Water–Propanol Mixtures

The transmission spectra of the phononic crystal made of the perforated steel block (see Figure 1c) were recorded using piezoelectric PZT transducers Panametrics V103-RB connected to the impedance analyzer Agilent4395A, between which the PnC was placed (see Section 2). The central hole was filled with a water–propanol mixture. In our work, we used the transmission measurement method. To the left and to the right of the phononic crystal structure, two piezoelectric transducers are located opposite to each other. The first acts as a transmitter, and the second as a receiver of an ultrasound waves. The transmitter sends an ultrasonic wave at a certain frequency, increasing with a certain step width and constant amplitude into the PnC structure. The receiver has a signal changed in intensity and phase as a result of the wave propagation through the structure and the interaction between ultrasound wave and PnC and liquid in cavity defect. A piezoelectric receiver converts this ultrasonic signal into an electrical signal, which is then compared with the input signal. The ratio of the output signal at the receiving to the input signal at the sending transmitter gives the S21 parameter. The measurement of the S21 parameter corresponds to the overall transmission behavior.

**Figure 5:**
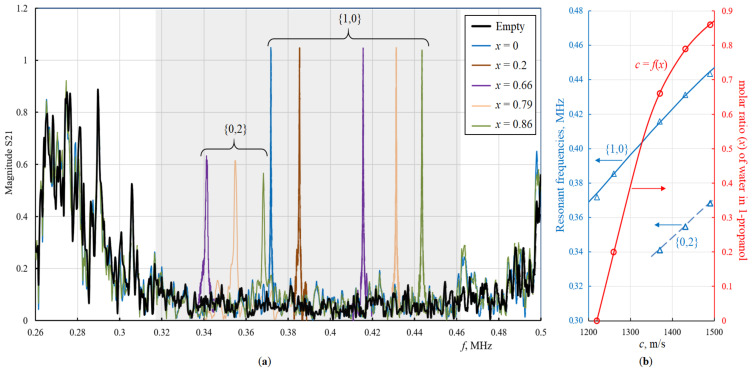
Experimental PnC transmission spectra of empty PnC and PnC with central hole filled by different compositions of 1-propanol + water mixtures (**a**), as well as dependence of the resonant frequencies of the {1,0} and {2,0} modes on the speed of sound of the mixture, determined by the molar ratio of water in 1-propanol (**b**), where blue lines show the theoretical results and the red curve shows the relationship between the composition of the mixture and speed of sound. The markers correspond to the experimental data.

Figure 5a shows the transmission spectrum of a phononic crystal, both empty and with a liquid-filled central cylindrical hole. The transmission spectrum of an empty PnC is shown as a thick black curve. It has an area of reduced transmission of acoustic waves, a bandgap, from 315 to 460 kHz. This area is highlighted in grey. When the central hole is filled with a liquid, resonant peaks appear in the band gap, which shift along the frequency axis to the right with an increased speed of sound of the liquid. The bandwidth taken at half maximum is 400 Hz (Q-factor is 1000). In Figure 5a, these peaks are shown in color for various mixtures of water and 1-propanol. The molar ratio (*x*) of water in 1-propanol, the speed of sound of the mixture (*c*) varies over a wide range. Figure 5b shows the dependence of the resonant frequencies of the radial and azimuthal vibrational modes on sound speed of the binary mixture (the left axis, blue lines show the theoretical calculation results, and the blue triangular markers correspond to the experiment points). The relationship between the composition of the mixture and the speed of sound (the right axis, the red curve) was taken from [25]. The red round markers correspond to experimental compositions.

To compare the azimuthal and radial eigenmodes on the same structure, the sound velocities of the mixtures were specially selected so that their resonance frequencies fall into the bandgap and correspond to the axisymmetric mode and to the azimuthal mode. As expected, the axisymmetric mode shows significantly higher and narrower peaks (Figure 5a). This is a direct consequence of analyzing real liquids, i.e., liquids having viscosity, and the direction of the displacement velocity vector close to the solid–liquid interface, which is perpendicular to the interface for the axisymmetric mode (see Figure 4). Based on the data for the sound velocity of a water–propanol mixture [25], it was not difficult to experimentally track and identify the position of the resonance peaks. The positions of the experimental peaks were in good agreement with theoretical predictions.

As noted earlier, the design of the phononic crystal was chosen in order to obtain the {1,0} mode within the PnC bandgap, which is true for a wide range of sound velocities in aqueous and alcohol solutions. As a result, isolated high-Q {1,0} peaks were obtained (see Figure 5a). Some of {0.2} modes for *x* = 0.66, 0.79 and 0.86 also fell into the band gap, but had a lower Q-factor. This allows us to distinguish them from the {1,0} peaks. Some of {0.2} modes for *x* = 0 and *x* = 0.2 are outside the band gap. They should be split and suppressed due to interaction with the eigenmodes of the steel lattice of the PnC and cannot be identified as individual isolated peaks. Since the low-frequency region of the spectrum to the left of the band gap is outside the optimal working frequency area of the PnC sensor as well as PZT transducers, they do not disturb the PnC sensor concept and have not been studied in detail.

For further studies, we used the radial mode in the central part of the bandgap of the phononic crystal since this mode provides the highest sensitivity and resolution of the PnC sensor structure.

### 3.3. Experiments with a Water–Fuel Emulsion

#### 3.3.1. Preparation and Properties of Water–Fuel Emulsion

To study water–fuel emulsion (WFE), a number of samples with water contents varying from 2.5 to 15% with a 2.5% increment were prepared from reference diesel fraction (Sigma-Aldrich, Darmstadt, Germany). The method of preparation strictly corresponded to the technology of making WFE trademarks, which are widely used worldwide as a fuel for diesel engines [34]. To disperse the samples during manufacturing, an ultrasonic cavitator was used for 20 min. To maintain the set temperature of the WFE during the process, a forced cooling of the sample was realized.

The particle size measurement of water in the WFE samples was carried out by Dynamic Light Scattering (DLS) in the backscatter detection mode at a θ = 175° detector angle using the Zetasizer Nano ZS particle characterization system (Malvern Instruments Ltd., Malvern, UK). More detailed information about the DLS method can be found in [34,35,36], see also Appendix C.

**Figure 6:**
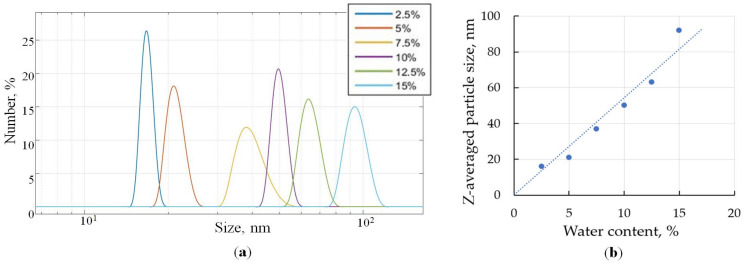
Particle size distribution for samples with different water content (**a**) and dependence of the z-averaged particle size on the water content in WFE (**b**).

Dynamic light scattering measurements were carried out for six samples with different water contents, the relative number distribution of particle sizes was extracted from the autocorrelation function for each sample by Zetasizer Nano ZS software v3.30 (Figure 6a).

The z-averaged particle size is proportional to the water content of the WFE (Figure 6b). In accordance with the graph, the size of all the particles in each sample is located in a fairly narrow range. This range is the narrowest for a sample with a water content of 2.5%. As the water content increases to 7.5%, the width of the range increases. For samples with a water content of 10% to 15%, this range corresponds approximately to a sample with a water content of 5%. It follows from the DLS data that the uniformity of the hydrodynamic diameters of water particles in a water–fuel mixture nonmonotonically depends on the water content; the most uniform emulsions are achieved for less than 5% or more than 10% of water.

**Figure 7:**
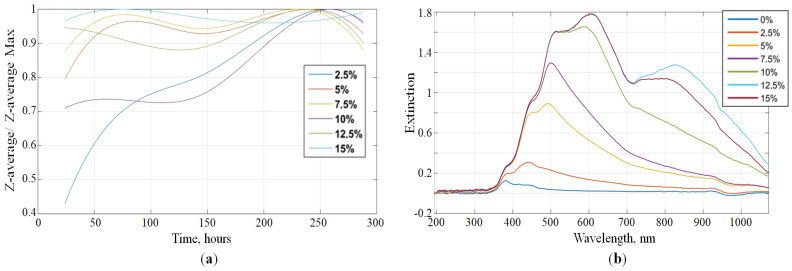
Graph of the relative change of the z-average time from the time of preparation (**a**) and spectral extinction of WFE samples with different water content (**b**).

To analyze the stability of WFE samples, dynamic light scattering measurements were repeated multiple times for 290 h after the time of preparation. The relative changes in z-averaged particle sizes are shown in Figure 7a.

The size of the water particles after the production increases for a while, and then stabilizes. The equilibrium of the system is reached. Following this, the particle size does not change significantly. The greatest increase in the size of water particles is inherent in samples with 10% and 15% water. Particles of water in the remaining samples increased slightly.

The size of the water particles after the production increases for a while and then stabilizes. The equilibrium of the system is reached. The greatest increase in the size of water particles was found in samples with 10% and 15% water. Particles of water in the remaining samples increased only slightly.

The WFE samples were studied by Vis-NIR spectrophotometry using the BLACK-Comet-SR spectrometer (StellarNet, Carlson Cir, FL, USA) and the external light source with a tungsten halogen lamp in the 350–1100 nm wavelength range. The samples were put into a quartz cuvette (1 mm light path). The results are shown in Figure 7b. For all the samples, a common pattern can be identified, which is typical for turbid media—several characteristic absorption bands combined with a strong scattering background. The wavelength at which the extinction maximum is reached increases with increasing water content in the WFE. For samples with a water content of 0% to 12.5%, extinction at a wavelength above 550 nm is directly proportional to the water content of the WFE samples.

#### 3.3.2. Measurement of the Transmission Spectra of Acoustic Waves through the PnC Sensor

**Figure 8:**
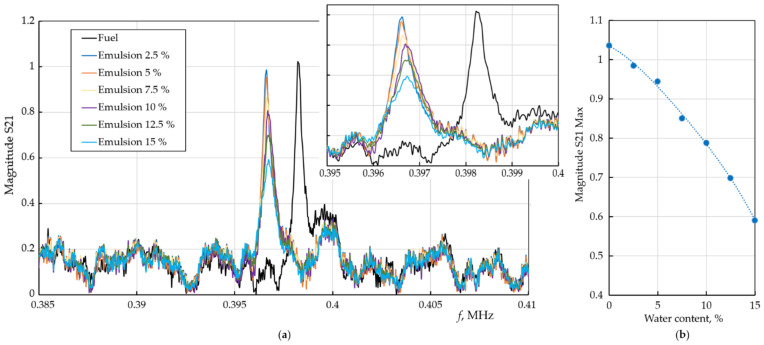
Experimental transmission spectra of the phononic crystal sensor with a cylindrical cavity defect filled with a water–fuel emulsion (**a**) and dependence of the amplitude (maximum intensity) of the transmission peaks on the water content (**b**). The dots show experimental points taken from the S21 measurements; the line is an interpolation.

The study of the transmission spectra of PnC with a defect filled with an emulsion was carried out similarly to the previous acoustic measurements of PnC structure. Since, as shown earlier, the axisymmetric mode has the higher Q, all measurements were performed using this mode (Figure 8a). The resonance peak corresponding to the homogeneous liquid (fuel) is the highest. When water was added, the peak of the emulsion gradually decreases in magnitude according to a nonlinear law (Figure 8b). For clarity, the inset in Figure 8a shows an enlarged area in a narrow frequency range around the resonant peaks.

The position of the peak on the frequency scale is associated with the chemical composition of the liquid. The change in chemical composition is associated with the addition of an emulsifier. This would lead to a shift of the resonance peak to the low-frequency region. Emulsions with different water content were prepared with the same emulsifier content, so the frequency of the resonance peak did not change significantly, only its amplitude changed.

## 4. Discussion

The phononic crystal sensor studied here is a two-dimensional periodic arrangement of cylindrical holes in a steel matrix. The PnC has been placed between two piezoelectric PZT transducers (Figure 1). The resonance frequencies correspond to two defect modes in the band gap (Figure 2) of this phononic crystal. They have been created by filling the central hole of the periodic structure with a liquid to be analyzed. The dependence of the resonant frequency of the sensor structure on the speed of sound of the liquid is linear inside the phononic crystal band gap. The measurement of mixtures of water and propanol as well as emulsions based on water and fuel are possible. A phononic crystal with a defect becomes a sensor that has high sensitivity and allows us to determine the concentration of a component in a homogeneous liquid mixture and in a heterogeneous liquid. The defect is a cylindrical resonator filled with the analyte and acts as measurement chamber. This causes a resonant peak. The resonant frequency of the cylindrical hole filled with liquid acts as measurement value. The defect is surrounded by the periodic structure, which provides a high acoustic contrast at the boundaries of the resonator. As the important result, a high Q-factor of the resonance peak can be achieved. The transmittance of the sensor at these frequencies does not suffer from significant attenuation of the signal. Variations in Q correspond to liquid properties. The resonance peak turns out to be isolated, since there are no vibrational eigenmodes of the solid-state structure within the band gap.

The geometry structure of the phononic crystal was designed in such a way that the eigenfrequency of the axisymmetric defect mode of a liquid-filled cylinder falls into the band gap for a wide range of values of the speed of sound of a liquid (Figure 3). The dependence of the resonant frequency of the defect mode on the speed of sound in central part of the forbidden zone is linear. Closer to the edges of the acoustic band gap, a nonlinearity is observed (see Figure 3b). Moreover, in the middle of the band gap, the behaviour of the defect is close to the behaviour of an ideal cylindrical resonator with completely reflecting walls. This suggests that at the center of the band gap, the defect mode is acoustically most isolated from the eigenmodes of the solid-state subsystem, while at the boundary of the band gap, the eigenfrequencies of the solid-state matrix begin to couple with the defect mode and shift its resonance frequency. The stronger the coupling, the larger the frequency shift and the smaller the Q. The frequency resolution is 4 Hz, based on the conservative assumption of 100 measurement points between the two frequencies at half maximum. The theoretical Q for 2D simulations is more then 10,000 for radial mode. Imperfections in fabrication and additional acoustic radiation losses at the edges of PnC structure mainly cause the difference between theory and experiment.

However, one cannot generally conclude the resolution of secondary values such as the concentration of a component in a liquid mixture. It depends on the relation between sound velocity of the mixture and the secondary value. It is known that the speed of sound of many real mixtures depends on the concentration in a nonlinear manner. Mixtures of alcohols and water even display a maximum, e.g., a PnC sensor, despite its high frequency resolution, becomes “deaf” in that small frequency range.

In addition to the axisymmetric defect mode, other peaks (Figure 3a) associated with the azimuthal modes of the central cylindrical resonator exist. The azimuthal modes have a lower Q and amplitude (Figure 5) and do not hinder the detection of the axisymmetric mode. The maximum liquid pressure of the azimuthal mode appears near the cylinder walls, while maximum liquid pressure of the axisymmetric mode emerges at the center. Consequently, the influence of the interphase boundaries for the azimuthal modes is much higher than for the axisymmetric one. Finally, due to the particle velocity profile of the axisymmetric resonant mode at the solid–liquid interface with a predominant normal direction, shear viscosity losses are insignificant and only bulk viscosity effects remain. The imaginary part of speed of sound, Im(*c*), depends on several other liquid parameters, that is the longitudinal and shear viscosity of the liquid, μ and η, the thermal conductivity, τ, as well as the density, ρ, the specific heat at constant pressure, *C*_p_, and the ratio of specific heats, γ [20]:

(3) c→c˜=Rec+iImc;Imc=πfρRecμ+43+γ−1Cpτ.

Consequently, the analysis of sound velocity and sound dissipation gives us access to more material-specific properties of a liquid mixture and their changes, e.g., during a technical or chemical process. The promise of using phononic crystals with a defect in the form of a liquid-filled cavity is due to access to those bulk properties of liquids (composition, concentrations, reaction kinetics, etc.). Fortunately, numerous articles have been published providing a large amount of experimental data. Ref. [37] is one of the most recent papers.

When a heterogeneous liquid is introduced into the defect cavity of a phononic crystal, the situation for the axisymmetric mode changes. The liquid contains a large number of scattering entities. Thermo-viscous losses significantly reduce the amplitude of the resonance peaks (Figure 8a). The higher the concentration of water in the water–fuel emulsion, the more interphase boundaries, the stronger the acoustic losses. Moreover, the experiments show that the dependence of the decrease in the amplitude of the peaks on the water concentration is nonlinear (Figure 8b). Further analysis is required, specifically regarding the droplet size distribution (Figure 6 and Figure 7). The change in the chemical composition of the liquid can be tracked by a shift in the resonance frequency of the peak (Figure 5), the change in the concentration of foreign-phase droplets can be monitored by a decrease in the peak amplitude (Figure 8). A deeper analysis of the properties of the analyte requires solving an inverse problem through Equation (3).

## 5. Conclusions

In this work, the properties of 2D phononic crystal steel/air structure of square symmetry with liquid-filled defect central hole were theoretically and experimentally investigated and their application as resonant acoustic sensors for the detection of bulk properties of homogeneous and heterogeneous liquids was shown.

The main conclusion of this work is that sensitivity of a phononic crystal sensor can be enhanced by the reduction in energy loss in the medium caused by liquid shear viscosity by utilization of the radial (axisymmetric) mode. The sensor does not require direct contact between the electrical and acoustic elements of the sensor and becomes a sensitive instrument for studying bulk properties of liquids even in harsh environment.

This is based on the following:

– The proposed phononic crystal with a liquid-filled defect provides a high-Q cavity resonance;
– In the proposed PnC sensor, the resonance frequency and the Q-factor are dependent on *volumetric* physical properties of the liquids;
– The dominant property that determines the resonance frequency is the *composition* of the liquid;
– The dominant factor usually determining the Q-factor are *viscous* losses at the solid–liquid interfaces;
– The *radial* (*axisymmetric*) vibration mode avoids shear displacement at the solid–liquid interface;
– Hence, the *radial* (*axisymmetric*) *mode* in cylindrical liquid-filled resonators gives access to the *highest* Q-factor (compared to other possible modes);
– The axisymmetric mode is, therefore, the key to an *enhanced* sensitivity of the PnC sensor to liquid analyte properties;
– The design of the PnC is the key to an optimal transduction of the plane wave into the radial cavity mode and backwards;
– Among others, changes in the chemical composition of the liquid or an increase in the concentration of droplets in heterogeneous liquids are examples of indirect sensor output values.

## Figures and Tables

**Figure A1:**
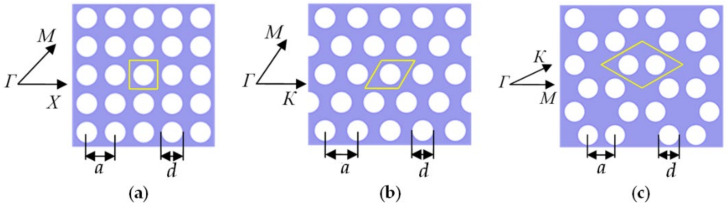
2D PnCs with square (**a**), hexagonal (**b**) and honeycomb (**c**) symmetries. The yellow rectangles represent the boundaries of unit cells.

**Figure A2:**
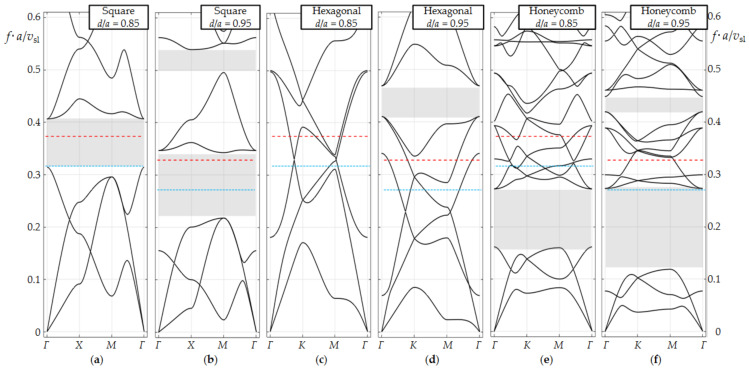
Band diagrams for 2D PnCs with square (**a**,**b**), hexagonal (**c**,**d**) and honeycomb (**e**,**f**) symmetries. Calculations are made for two ratios: *d*/*a* = 0.85 (**a**,**c**,**e**) and 0.95 (**b**,**d**,**f**). Red and blue dotted lines correspond to liquid pressure {1,0} mode resonances in ideal resonator (the diameter of the IR is equal to the diameters of the PnC holes) for water and propanol at the temperature of 20 °C. Here, PnC and IR act independently; other modes of IR are not shown so as not to overload the figure.

**Figure A3:**
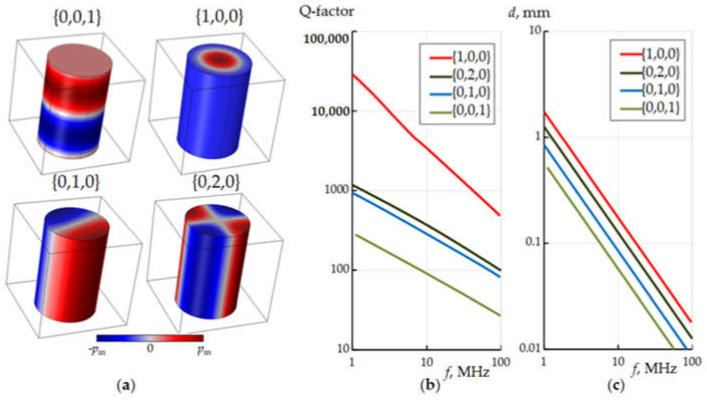
Resonant modes in a liquid-filled cylindrical cavity and their Q-factors (**a**). Dependence of the Q-factor of the eigenmodes on the resonance frequency (**b**) and the corresponding values of the inner diameter of the cylindrical resonator (**c**). Calculations are made for water at 20 °C. When calculating the Q-factor, only thermal-viscous losses at the solid–liquid interface and in the bulk of the liquid were taken into account.

**Figure A4:**
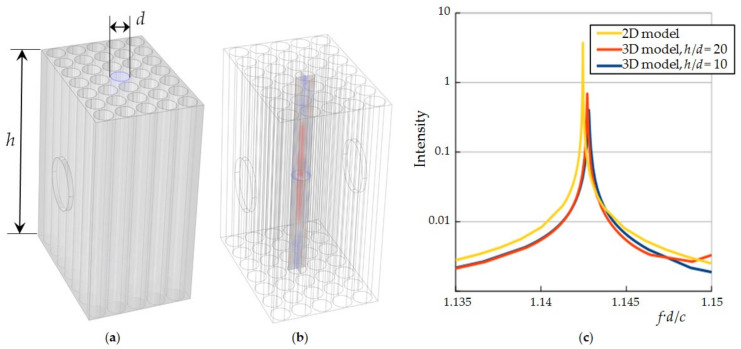
3D model of a two-dimensional PnC (**a**) and simulation results of the pressure distribution in the middle liquid-filled cylinder for the {1,0,0} mode (**b**) and its resonant peak for different heights of the PnC structure, as well as in comparison with the 2D model (**c**). A real two-dimensional PnC has boundaries at its height. At these boundaries, the PnC breaks off; here, acoustic energy losses for radiation are possible. Radiation losses reduce the resonance Q-factor and sensitivity.

## Data Availability

Not applicable (The original data obtained by the authors were used in the work. For additional information, please contact the authors).

## Appendix A. Details of PnC Sensor Numerical Modelling

The numerical calculation and analysis of the frequency characteristics of phononic crystal was performed on the basis of the equation for the propagation of mechanical waves in an elastic medium [32]:

(A1) $$\rho_{S}\frac{\partial^{2}u_{i}\left(r,t\right)}{\partial t^{2}}=\sum_{j}\frac{\partial T_{ij}}{\partial x_{j}},\;\;i,j=1,2,3,$$

where *u_i_*(**r**, *t*) are elastic displacement field components; *T_ij_* are stress tensor components; ρ_S_ is the solid matrix density; **r** = (x, y, z) is vector coordinates; *t* is time.

For nonpiezoelectric materials,

(A2) $$T_{ij}=\sum_{k}\sum_{l}c_{ijkl}^{}S_{kl},\;\;i,j,k,l=1,2,3,$$

where *c_ijkl_* are elastic tensor components; *S_kl_* are strain tensor components.

For piezoelectric materials,

(A3) $$T_{ij}=\sum_{k}\sum_{l}c_{ijkl}^{E}S_{kl}-\sum_{k}e_{kij}^{}E_{k},\;\;i,j,k,l=1,2,3,$$

where *c^E^_ijkl_* are elastic tensor components determined at a constant electric field **E**; *e_kij_* are components of the piezoelectric tensor.

The deformation at each point is determined by the equation

(A4) $$S_{ij}=\frac{1}{2}\left(\frac{\partial u_{i}}{\partial x_{j}}+\frac{\partial u_{j}}{\partial x_{i}}\right),\;\;i,j=1,2,3.$$

Considering that a phononic crystal is a periodic structure, the Bloch theorem was used to find the eigenresonance solutions, according to which the displacement vector can be represented as the product of a propagating wave and a periodic function of a phononic crystal structure:

(A5) $$u\left(r,k\right)=u_{k}\left(r\right)\exp\left(-ikr\right),\;\;u_{k}\left(x,y,z\right)=u_{k}\left(x+a,y+a,z\right),$$

where **u***_k_*(**r**) is a periodic function of **r**; **k** is wave vector.

The specific features of the acoustic spectra of studying structure with a liquid filler are associated with resonances of the liquid pressure in cylindrical hole. The change in pressure can be described in the form of a wave equation for given boundary conditions. Resonant modes can be found by solving the problem of finding eigenfrequencies for acoustic modes in a cylindrical hole. The main equation for a pressure wave with harmonic solutions is the Helmholtz equation, which can be represented as follows:

(A6) $$\nabla \left(-\frac{1}{\rho_{L}}\nabla p\right)-\frac{\omega^{2}p}{\rho_{L}c^{2}}=0,$$

where ρ_L_ is liquid density; ω is circular frequency; *c* is the speed of sound in a liquid; *p* is pressure.

The conditions at the solid–liquid interfaces are:

(A7) $$F=-n_{s}p;\;\;\left(n_{f}\cdot u\right)\omega^{2}=-n_{f}\left(-\frac{1}{\rho_{L}}\nabla p+q\right),$$

where **n***_s_* is the normal vector directed from the solid-state body; **F** is the force per unit area representing the load on the cylinder walls, **n***_f_* is the normal vector directed from the volume of the liquid; **u** is the vector of mechanical displacement in a solid; **q** is the acceleration vector imparted to the fluid.

To calculate viscosity losses, the Navier–Stokes equation should be used instead of the Helmholtz equation [33]:

(A8) $$\omega^{2}v+\left[\frac{c^{2}}{\gamma }-i\omega \frac{\eta }{\rho }\left(\frac{3}{4}+\frac{\mu }{\eta }\right)\right]\nabla \left(\nabla \cdot v\right)+i\omega \frac{\eta }{\rho }\nabla \times \nabla \times v=\frac{i\omega \beta c^{2}}{\gamma }\nabla T;$$

(A9) $$\gamma \frac{\tau }{\rho C_{p}}\nabla^{2}T+i\omega T=\frac{\left(\gamma -1\right)}{\beta }\nabla \cdot v,$$

where **v** is the velocity vector of the liquid; *T* is the temperature; γ is the coefficient of specific heat, τ is the thermal conductivity; ρ is the density; *C*_p_ is the specific heat at constant pressure; β is the volumetric compressibility; η is the shear viscosity; μ is the bulk viscosity; *c* is the speed of sound.

The described Equations (A1)–(A4), (A6), (A8) and (A9) together with the boundary conditions (A5) and (A7) are sufficient for calculating the propagation of acoustic waves in solid and liquid media. On their basis, simulations were numerically carried out in this article by the finite element method.

## Appendix B. Aspects of PnC Sensor Configuration and Sensitivity

Before choosing a specific phononic crystal sensor design, we theoretically investigated various configurations of 2D PnCs, with square, triangular and honeycomb symmetries (Figure A1) and filling factors (Figure A2).

We found that the square lattice is the most suitable solution in terms of simplicity and compatibility to the following challenge: wide bandgap with the resonant frequencies of the radial mode of a liquid-filled cavity defect of the same size as regular ones for a wide range of sound velocities in aqueous and alcohol solutions. The use of triangular or honeycomb symmetries instead of square symmetries does not provide any significant advantages for our sensor application. A change in the filling factor (*FF*) leads to a change in the band gap width. The choice of *FF* was determined by the fact that a decrease in the *FF* leads to a decrease in the band gap (and consequently to a decrease in the operating frequency range of the sensor). Furthermore, an increase in the *FF* makes the manufacturing process more involved.

A sensor design based on a liquid-filled cavity resonator requires a co-operative action between cavity and PnC. On the one hand, the cavity resonator needs a high Q. On the other hand, the resonance must be measurable from the outside. In other words, some acoustic energy becomes “lost” to the environment. Since the electroacoustic transducers generate and receive plane waves, a dedicated mode conversion is required.

This paper concentrates on the increase in the effective (or reduced) sensitivity of the sensor, i.e.,

(A10) $$S_{red}=\frac{\Delta f}{f_{res}\times f_{HBHM}},$$

where Δf is the frequency shift caused by the analyte, e.g., the concentration of a component in a mixture, $f_{res}$ is the resonance frequency employed; in our case, the resonance frequency of a cavity mode, $f_{HBHM}$ is the half the frequency difference at half maximum.

It becomes obvious that the effective sensitivity can be increased by a reduction in $f_{HBHM}$. This brings us to liquid viscosity. This value has been neglected quite often. It is justified in several cases to simplify the calculation. However, at resonance, the Q also amplifies loss mechanisms, which appear in volume, specifically those caused by liquid viscosity. The most important part of the paper deals with that problem and introduces the radial mode to eliminate liquid viscosity losses, and to be more exact regarding losses caused by liquid shear viscosity. Bulk viscosity of the liquid can generally not be avoided. This value is smaller than shear viscosity; however, it still is the most important loss mechanism at liquid resonance.

The primary value of the sensor is the (complex) resonance frequency, which takes us directly to liquid sound velocity and viscosity. To determine secondary values such as the concentration of a component in a mixture requires preliminary knowledge. Something similar applies to density as one parameter determining acoustic impedance of the liquid and hence the reflection coefficient at the PnC–liquid interface. In the ideal case, density is just a secondary value related to peak amplitude. Liquid viscosity is dominant. However, both speed of sound and density are material properties. Regarding uncertainty, this is determined by the Q and the quality of the measurement setup.

The Q-factors of the liquid-filled cavity eigenmodes were calculated taking into account the thermal and viscous damping mechanisms in the liquid volume and at the liquid–solid interfaces (Figure A3b). The red, black, blue and green colours of the lines correspond to the first radial, second azimuthal, first azimuthal and first longitudinal modes (Figure A3b,c). The relationship between the diameter and the resonant frequency for each mode is shown in Figure A3c. In Figure A3, we have added a third index in the designation of the eigenmodes of the cylindrical resonator to take into account the longitudinal mode (this becomes important for the 3D case).

The highest Q-factor of the radial mode can be explained by the specific pressure distribution at which surface friction is negligible and surface losses are significantly reduced.

It is important to note that the finite size in all directions of the 3D PnC influences the transmission characteristics. At these boundaries, the PnC breaks off; here, acoustic energy losses due to radiation are possible. Radiation losses reduce the resonance Q-factor and sensor sensitivity. Most important for the sensor resolution are the conditions at the edges of the structure. Specifically, the finite height of the PnC is insignificant with regard to the position of the resonant peaks of defect and the band gap width, since it is 10 times larger than the diameter of the holes and the PnC can be considered as quasi-two-dimensional. Figure A4 shows the results of simulating the Q-factor of the resonance peak of a PnC as a function of its height. Here, the abscissa is a dimensionless value obtained by multiplying the frequency by the diameter of the cylinder and dividing by the speed of sound of the liquid.

At the edges of the phononic structure, radiation losses were taken into account as boundary conditions. The higher the structure, the higher the Q-factor. However, the solution to the problem of radiation losses will further be investigated.

## Appendix C. Aspects of Dynamic Light Scattering Measurements

The Dynamic Light Scattering technique relies on the measurement and time-correlation analysis of the fluctuations of the scattered laser radiation intensity as a result of Brownian motion exhibited by dispersed particles—in this case, water droplets in water–fuel emulsions [34,35]. The autocorrelation function of the scattered radiation depends on the translational diffusion coefficient of a droplet *D*_eff_ [35]:

(A11) $$g\left(\tau \right)=Ae^{-D_{\mathrm{eff}}q^{2}\tau }+B;\;\;q=\frac{4\pi n}{\lambda }\sin\left(\frac{\theta }{2}\right),$$

where *A* is the amplitude of correlation, *B* is the baseline, *q* is the scattering vector, *n* is the refractive index of the dispersing medium, λ is the wavelength of the laser. The hydrodynamic diameter of a particle *D*_h_ can be found from the Stokes−Einstein relationship [36]:

(A12) $$D_{\mathrm{eff}}=\frac{k_{B}T}{3\pi \eta D_{h}},$$

where *k*_B_ is the Boltzmann constant, *T* is the absolute temperature of a sample, η is the solvent viscosity. The hydrodynamic diameter is the diameter of a hypothetical sphere that diffuses in the medium at the same rate as the particle under investigation [36]. Because water droplets in water–fuel emulsions could be considered spherical, hereafter the hydrodynamic diameter of these droplets will be referred to as the particle size.
